# Efficacy and Safety of Anti–Vascular Endothelial Growth Factor Monotherapies for Neovascular Age-Related Macular Degeneration: A Mixed Treatment Comparison

**DOI:** 10.3389/fphar.2021.797108

**Published:** 2021-12-21

**Authors:** Yun Zhang, Sheng Gao, Xun Li, Xi Huang, Yi Zhang, Tiancong Chang, Zhaolun Cai, Meixia Zhang

**Affiliations:** ^1^ Department of Ophthalmology, West China Hospital, Sichuan University, Chengdu, China; ^2^ Research Laboratory of Macular Disease, West China Hospital, Sichuan University, Chengdu, China; ^3^ Department of Gastrointestinal Surgery, West China Hospital, Sichuan University, Chengdu, China

**Keywords:** anti–vascular endothelial growth factor monotherapy, neovascular age-related macular degeneration, efficacy, safety, mixed treatment comparison

## Abstract

**Background:** We aimed to evaluate the comparative efficacy and safety of anti–vascular endothelial growth factor (anti-VEGF) monotherapy to identify its utilization and prioritization in patients with neovascular age-related macular degeneration (nAMD).

**Methods:** Eligible studies included randomized controlled trials comparing the recommended anti-VEGF agents (ranibizumab, bevacizumab, aflibercept, brolucizumab, and conbercept) under various therapeutic regimens. Outcomes of interest included the mean change in best-corrected visual acuity (BCVA), serious adverse events, the proportion of patients who gained ≥15 letters or lost <15 letters in BCVA, the mean change in central retinal thickness, and the number of injections within 12 months.

**Results:** Twenty-seven trials including 10,484 participants and eighteen treatments were identified in the network meta-analysis. The aflibercept 2 mg bimonthly, ranibizumab 0.5 mg T&E, and brolucizumab 6 mg q12w/q8w regimens had better visual efficacy. Brolucizumab had absolute superiority in anatomical outcomes and a relative advantage of safety, as well as good performance of aflibercept 2 mg T&E. The proactive regimens had slightly better efficacy but a slightly increased number of injections versus the reactive regimen. Bevacizumab had a statistically non-significant trend toward a lower degree of efficacy and safety.

**Conclusion:** The visual efficacy of four individual anti-VEGF drugs is comparable. Several statistically significant differences were observed considering special anti-VEGF regimens, suggesting that brolucizumab 6 mg q12w/q8w, aflibercept 2 mg bimonthly or T&E, and ranibizumab 0.5 mg T&E are the ideal anti-VEGF regimens for nAMD patients. In the current landscape, based on the premise of equivalent efficacy and safety, the optimal choice of anti-VEGF monotherapies seems mandatory to obtain maximal benefit.

## 1 Introduction

Age-related macular degeneration (AMD) is a progressive and degenerative retinal disease that causes severe and irreversible vision loss in people older than 50 years (Flaxel et al., 2020). As populations age, the worldwide prevalence of AMD increases annually, with an expected increase to 288 million in 2040 (Wong et al., 2014). Late-stage AMD is characterized by the gradual loss of central vision due to geographical atrophy or by the rapid loss of central vision due to neovascularization (Mehta et al., 2018). Choroidal neovascularization (CNV) is the typical pathological feature of neovascular AMD (nAMD), which is controlled by various growth factors, such as vascular endothelial growth factor (VEGF), and typified by an anomalous angiogenic process (Bressler, 2009). Numerous studies have confirmed the efficacy and safety of anti-VEGF therapy, and published guidelines currently recommend anti-VEGF therapy as the first-line treatment for patients with nAMD (23 January 2018; Flaxel et al., 2020; Yeung et al., 2021), including ranibizumab (Lucentis, Genentech Inc.), bevacizumab (Avastin, Genentech Inc.), aflibercept (Eylea, Regeneron Pharmaceuticals, Inc.), conbercept (Lumitin; Chengdu Kanghong, Inc.) and, recently, brolucizumab (Beovu, Novartis, Inc.) (Schmidt-Erfurth et al., 2014; Flaxel et al., 2020).

There are currently large numbers of therapeutic regimens of anti-VEGF therapy with different drugs, dosages, and therapeutic strategies, while controversies remain regarding the ideal drug and optimum therapeutic strategy (Singer et al., 2012; Ogura et al., 2015; Solomon et al., 2019). However, it is difficult to acquire comparative efficacy and safety profiles from current trials due to the lack of head-to-head trials. Meanwhile, several problems in published meta-analyses, such as indiscriminate merger, inaccurate dosage distinction, and redundancy inclusion, remain to be resolved with more precise analysis (Ye et al., 2020; Zhao et al., 2021). Here, we report comprehensive monotherapy-based mixed treatment comparisons (Bayesian network meta-analysis) of efficacy (visual acuity and anatomical structure), safety, and therapeutic frequency in patients receiving anti-VEGF monotherapy, which can overcome the shortage of direct comparison trials and combine direct and indirect data to compare two or more interventions simultaneously to identify the utilization and prioritization of anti-VEGF monotherapies in nAMD patients.

## 2 Methods

### 2.1 Protocol and Registration

The study protocol is registered in PROSPERO (http://www.crd.york.ac.uk/PROSPERO/; Registration number: CRD42018103227). The study was structured according to the Preferred Reporting Items for Systematic Reviews and Meta-Analyses extension for network meta-analysis (Hutton et al., 2015).

### 2.2 Information Sources and Search Strategy

The electronic PubMed, Embase, and Cochrane Library databases were systematically searched. The search terms included “macular degenerations,” “age-related maculopathy,” “age-related macular degenerations,” “ranibizumab,” “bevacizumab,” “aflibercept,” “brolucizumab,” “conbercept,” “angiogenesis inhibitors,” and “verteporfin.” The detailed search strategies are presented in Supplementary Table S1. The reference lists of the relevant studies and review articles were also checked. The last search was carried out on April 16th, 2021.

### 2.3 Eligibility Criteria

The detailed eligibility criteria were summarized using the PICOS approach (patient, intervention, comparison, outcome, and study design type).

#### 2.3.1 Patients and Comparison of Interventions

We included randomized controlled trials (RCTs) that compared two or more of the following treatment strategies (placebo and different anti-VEGF monotherapy regimens) for patients with nAMD. To maximize the clinical significance of our study, only the anti-VEGF agent doses that were approved or recommended by the guidelines (Flaxel et al., 2020) were analyzed, and the anti-VEGF agent doses included ranibizumab 0.5 mg, aflibercept 2 mg, bevacizumab 1.25 mg, brolucizumab 3 and 6 mg, and conbercept 0.5 mg. Although bevacizumab was used off-label, its effects were confirmed by several tests. So, bevacizumab was included in this study. To obtain more indirect evidence, we also included IVR 0.5 mg with PDT and standard PDT therapy to provide a more complete network closed loop.

We strictly distinguished each therapy regimen and did not combine any different treatment strategies to obtain the most accurate and recommended statistical evidence for clinical practice. We excluded drugs and regimens that had been proven to be ineffective or that were not recommended by the guidelines, such as pegaptanib, ranibizumab 0.3 mg, aflibercept 0.5 mg, and triamcinolone acetonide. We also excluded drugs and regimens that lacked available indirect evidence. There were no restrictions in terms of age, ethnic distribution, or sex.

#### 2.3.2 Outcomes

Trials should contain at least one of the primary outcomes. The primary outcomes included the mean change in BCVA from the baseline and the number of serious adverse events (SAEs), including serious ocular and serious systemic (or non-ocular) adverse events. The secondary outcomes included 1. the proportion of patients who gained ≥15 letters in BCVA from the baseline; 2. the proportion of patients who lost <15 letters in BCVA from the baseline; 3. the mean change in central retinal thickness (CRT) from the baseline; and 4. the mean number of injections. All the outcomes were analyzed at 12 months.

#### 2.3.3 Study Design

Only published RCTs without date or language restrictions were included in the network meta-analysis. We excluded conference abstracts, letters, reviews, case reports, or non-clinical studies without available data. Studies with overlapping data or insufficient data were excluded after a reasonable attempt at contacting the corresponding authors.

### 2.4 Study Selection and Data Collection

Two reviewers independently screened and selected the studies and extracted the relevant data from the included studies (Higgins et al., 2011). All study characteristics were summarized in the same standardized collection form by two reviewers. The data included study characteristics, patient characteristics, data needed for quality assessment, and outcomes. Disagreements were resolved by discussion with a third reviewer.

### 2.5 Risk of Bias

The risk of bias of individual studies was assessed using Cochrane Collaboration’s tool for RCTs (Higgins et al., 2011) in terms of sequence generation and allocation concealment (selection bias), blinding of participants and researchers (performance bias), blinding of outcome assessment (detection bias), incomplete outcome data (attrition bias), selective reporting (reporting bias), and other random biases. Disagreements were resolved in discussion with a third reviewer who acted as an arbitrator.

### 2.6 Data Synthesis and Statistical Analysis

The network meta-analyses using Bayesian methods (Lumley, 2002) were performed using Stata 14 (Stata Corp, College Station, TX, United States), JAGS, and R version 4.1.0. The network plots were generated by using Igraph tools in Hiplot (2021)(https://hiplot.com.cn). The I^2^ test was used to quantify the effect of heterogeneity in the model. The heterogeneity was assessed as high if I^2^ > 50%, and the random effects model was used. Treatment effects were expressed as the risk ratio (RR) and weighted mean difference (WMD) for dichotomous data and continuous (mean difference) data, respectively. We used 95% credible intervals (CrIs) to estimate the network meta-analyses. The node-splitting method was used to calculate the inconsistency of the model to assess the consistency. A *p* value < 0.05 indicated a significant inconsistency (Dias et al., 2010). We estimated the potential ranking probability of treatments by calculating the surface under the cumulative ranking curve (SUCRA) for each intervention (Salanti et al., 2011). The SUCRA value ranged from 0 to 1, and the treatments with higher SUCRA values were considered to have better efficacy. Network plots were drawn to present the comparisons of the interventions across trials to ensure whether a network meta-analysis was feasible. Trials were excluded if the investigated treatments could not be connected by other treatments.

## 3 Results

### 3.1 Study Selection and Characteristics of Included Studies

**FIGURE 1:**
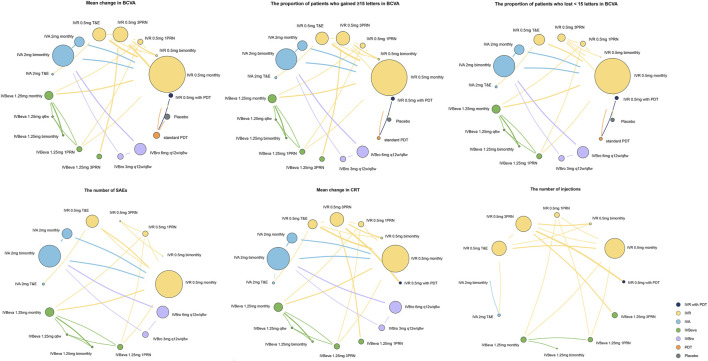
Network plots of comparisons for treatment-based network meta-analyses. Each circular node represents a type of treatment. The circle size is proportional to the total number of patients. The width of lines is proportional to the number of studies performing head-to-head comparisons. A total of 27 trials were analyzed.

We identified 4,326 potentially relevant studies. Twenty-seven RCTs (25 trials) that met the inclusion criteria were included in the network analysis (Heier et al., 2006; Rosenfeld et al., 2006; Brown et al., 2009; Kaiser, 2009; Martin et al., 2011; El-Mollayess et al., 2012; Heier et al., 2012; Larsen et al., 2012; Krebs et al., 2013a; Krebs et al., 2013b; Busbee et al., 2013; Kodjikian et al., 2013; Lushchyk et al., 2013; Wykoff et al., 2015; Schauwvlieghe et al., 2016; Weingessel et al., 2016; Feltgen et al., 2017; Haga et al., 2018; Silva et al., 2018; Amarakoon et al., 2019; Dugel et al., 2019; Gillies et al., 2019; Kertes et al., 2019; Wang et al., 2019; Lopez Galvez et al., 2020). Overall, a total of 10,484 patients with nAMD were involved in the study. The included trials compared the following 18 interventions: intravitreal aflibercept 2 mg monthly (IVA 2 mg monthly); IVA 2 mg monthly for 3 months, followed by IVA 2 mg bimonthly (IVA 2 mg bimonthly); IVA 2 mg treat-and-extend (IVA 2 mg T&E); intravitreal bevacizumab 1.25 mg monthly (IVBeva 1.25 mg monthly); IVBeva 1.25 mg every 6 weeks (IVBeva 1.25 mg q6 weekly); IVBeva 1.25 mg bimonthly; IVBeva 0.5 mg as needed after one initial injection (1 pro re nata (PRN) (IVBeva 1.25 mg 1PRN); IVBeva 1.25 mg as needed after three initial monthly injections (IVBeva 1.25 mg 3PRN); intravitreal brolucizumab (IVBro) 3 mg every 12 weeks (q12w) and were interval adjusted to every 8 weeks (q8w) if disease activity was present (IVBro 3 mg q12w/q8w); IVBro 6 mg q12w/q8w; intravitreal ranibizumab 0.5 mg monthly (IVR 0.5 mg monthly); IVR 0.5 mg bimonthly; IVR 0.5 mg 1PRN; IVR 0.5 mg 3PRN; IVR 0.5 mg T&E; standard dose of PDT 1 day after IVR 0.5 mg at the baseline, followed by two monthly IVR injections and IVR 0.5 mg as needed thereafter (IVR 0.5 mg with PDT); standard PDT at the baseline followed by PDT as needed (standard PDT); and placebo. Conbercept was excluded because there were no head-to-head RCTs available to connect to the node graph of the network. The network plots of all analyzed comparisons are shown in Figure 1. The characteristics of the included studies are summarized in Table 1. The literature screening process is shown in Supplementary Figure S1.

**TABLE 1:** Efficacy outcome: ①Mean change in BCVA; ②The number of SAEs; ③The proportion of patients who gained ≥15 letters in BCVA; ④The proportion of patients who lost <15 letters in BCVA; ⑤Mean change in CRT; ⑥The mean number of injections. Abbreviations: BCVA, best-corrected visual acuity; CRT, central retinal thickness; IVA, intravitreal aflibercept; IVBeva, intravitreal bevacizumab; IVBro, intravitreal brolucizumab; IVR, intravitreal ranibizumab; PDT, photodynamic therapy; PRN, pro re nata; Q6weekly, every six weeks; Q12w/q8w: every 12 weeks and were interval adjusted to every 8 weeks if disease activity was present; T&E, treat-and-extend.

Author, year	Register number	Drugs	Dose	Therapeutic regimens	Sample size	Mean age	Outcomes
Weingessel et al. (2016)	—	IVR	0.5 mg	3PRN	16	81.1	①⑤⑥
		IVR/PDT	0.5 mg/standard	IVR 0.5 mg with PDT	14	83.3	
Schauwvlieghe et al. (2016)	NTR1704^b^	IVBeva	1.25 mg	Monthly	161	79	①②③④⑤
		IVR	0.5 mg	Monthly	166	78	
Krebs et al. (2013a)	NCT01570608^a^	IVR	0.5 mg	3PRN	24	77.71	①⑤⑥
		IVR/PDT	0.5 mg/standard	IVR 0.5 mg with PDT	20	80.25	
Krebs et al. (2013b)	NCT00710229^a^	IVR	0.5 mg	3PRN	163	77.6	①③⑤⑥
		IVBeva	1.25 mg	3PRN	154	76.7	
Kodjikian et al. (2013)	NCT01170767^a^	IVR	0.5 mg	3PRN	183	76.68	①③⑤⑥
		IVBeva	1.25 mg	3PRN	191	79.62	
Larsen et al. (2012)	NCT00433017^a^	IVR (Sham PDT)	0.5 mg	3PRN	133	75.5	①③④⑤⑥
		IVR/PDT	0.5 mg/standard	IVR 0.5 mg with PDT	122	76.8	
Heier et al. (2012) (VIEW1)	NCT00509795^a^	IVA	2 mg	Monthly	304	77.7	①②③④⑤
		IVA	2 mg	Bimonthly	301	77.9	
		IVR	0.5 mg	Monthly	304	78.2	
Heier et al. (2012) (VIEW2)	NCT00637377^a^	IVA	2 mg	Monthly	309	74.1	①②③④⑤
		IVA	2 mg	Bimonthly	306	73.8	
		IVR	0.5 mg	Monthly	291	73.0	
Martin et al. (2011)	NCT00593450^a^	IVR	0.5 mg	Monthly	301	79.2	①②③④⑤⑥
		IVR	0.5 mg	1PRN	298	78.4	
		IVBeva	1.25 mg	Monthly	286	80.1	
		IVBeva	1.25 mg	1PRN	300	79.3	
Kaiser. (2009)	NCT00121407^a^	PDT	Standard	1PRN	244	79	①
		Placebo			120	79	
Brown et al. (2009)	NCT00061594^a^	PDT (Sham IVR)	Standard	1PRN	143	77.7	①③④
		IVR (Sham PDT)	0.5 mg	Monthly	140	76	
Rosenfeld et al. (2006)	NCT00056836^a^	IVR	0.5 mg	Monthly	240	77	①③④
		Placebo (Sham IVR)	—	—	238	77	
Heier et al. (2006)	NCT00056823^a^	IVR/PDT	0.5 mg/standard	IVR 0.5 mg with PDT	106	74.7	①③④
		PDT (sham IVR)	Standard	1PRN	56	73.0	
Silva et al. (2018)	NCT01948830^a^	IVR	0.5 mg	T&E	323	75.2	①②③④⑤⑥
		IVR	0.5 mg	Monthly	327	75.3	
Wykoff et al. (2015)	NCT01648292^a^	IVR	0.5 mg	Monthly	20	77	①②③⑤
		IVR	0.5 mg	T&E	40	77	
Lushchyk et al. (2013)	NTR1174^b^	IVBeva	1.25 mg	Monthly	64	76.5	①②③④⑤
		IVBeva	1.25 mg	Q6 weekly	63	77.4	
		IVBeva	1.25 mg	Bimonthly	64	78.1	
Busbee et al. (2013)	NCT00891735^a^	IVR	0.5 mg	Monthly	275	78.8	①③④⑤⑥
		IVR	0.5 mg	3PRN	275	78.5	
EI-Mollayess et al. (2012)	—	IVBeva	1.25 mg	1PRN	60	76.8	①②③④⑤
		IVBeva	1.25 mg	Q6 weekly	60	76.8	
Kertes et al. (2019)	NCT02103738^a^	IVR	0.5 mg	T&E	287	78.9	①②③④⑥
		IVR	0.5 mg	Monthly	293	78.8	
Gillies et al. (2019)	NCT02130024^a^	IVR	0.5 mg	T&E	142	76.6	①②③④⑤⑥
		IVA	2 mg	T&E	139	78.7	
Amarakoon et al. (2019)	NTR1174^b^	IVBeva	1.25 mg	Monthly	60	77.6	①②③④⑤⑥
		IVBeva	1.25 mg	Bimonthly	60	79.1	
Dugel et al. (2019) (HAWT)	NCT02307682^a^	IVBro	3 mg	Q12w/q8w	358	76.7	①②③④⑤
		IVBro	6 mg	Q12w/q8w	360	76.7	
		IVA	2 mg	Bimonthly	360	76.2	
Dugel et al. (2019) (HARRIER)	NCT02434328^a^	IVBro	6 mg	Q12w/q8w	370	74.8	①②③④⑤
		IVA	2 mg	Bimonthly	369	75.5	
Lopez Galvez et al. (2020)	EudraCT number 2012-003431-37^c^	IVR	0.5 mg	Bimonthly	103	77.9	①③⑤⑥
		IVR	0.5 mg	T&E	99	77.9	
		IVR	0.5 mg	3PRN	104	77.9	
Wang et al. (2019)	NCT02810808^a^	IVR	0.5 mg	1PRN	45	69.7	①②③④⑤
		IVR	0.5 mg	3PRN	49	70.0	
Feltgen et al. (2017)	EudraCT number 2009-017324-11^c^	IVR	0.5 mg	Bimonthly	20	79.0	①②③⑤⑥
		IVR	0.5 mg	3PRN	20	81.0	
Haga (et al. 2018)	UMIN ID 000014946^d^	IVA	2 mg	T&E	21	75.5	②③④⑤⑥
		IVA	2 mg	Bimonthly	20	78.5	

### 3.2 Mean Change in BCVA

**FIGURE 2:**
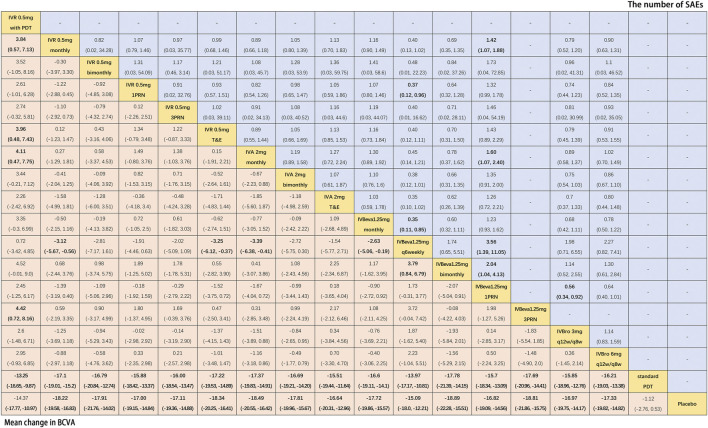
Pairwise comparisons of the network meta-analyses. Comparison of the included interventions: Weighted mean differences (95% CrI) are for the mean change in BCVA, and risk ratios (95% CrI) are for the number of SAEs. Bold cells are significant. For the mean change in BCVA, weighted mean differences <0 favor column-defining treatment. For the number of SAEs, a risk ratio <1 favors column-defining treatment.

Twenty-six trials comparing eighteen interventions in terms of mean change in BCVA at 12 months from the baseline were examined (Heier et al., 2006; Rosenfeld et al., 2006; Brown et al., 2009; Kaiser, 2009; Martin et al., 2011; El-Mollayess et al., 2012; Heier et al., 2012; Larsen et al., 2012; Krebs et al., 2013a; Krebs et al., 2013b; Busbee et al., 2013; Kodjikian et al., 2013; Lushchyk et al., 2013; Wykoff et al., 2015; Schauwvlieghe et al., 2016; Weingessel et al., 2016; Feltgen et al., 2017; Silva et al., 2018; Amarakoon et al., 2019; Dugel et al., 2019; Gillies et al., 2019; Kertes et al., 2019; Wang et al., 2019; Lopez Galvez et al., 2020). Figure 2 shows the results based on a Bayesian network meta-analysis that combines direct and indirect comparisons. Compared with placebo and standard PDT therapy, all anti-VEGF monotherapies were clearly superior in terms of the mean change in BCVA at 12 months. Compared with IVR 0.5 mg with PDT, IVR 0.5 mg monthly (WMD, 3.84 [95% CrI, 0.57–7.13]), IVR 0.5 mg T&E (WMD, 3.96 [95% CrI, 0.48–7.43]), IVA 2 mg monthly (WMD, 4.11 [95% CrI, 0.47–7.75]), and IVBeva 1.25 mg 3PRN (WMD, 4.42 [95% CrI, 0.72–8.16]) were associated with a statistically significant superiority in terms of mean change in BCVA. Compared with IVBeva 1.25 mg q6 weekly, IVR 0.5 mg monthly (WMD, 3.12 [95% CrI, 0.56–5.67]), IVR 0.5 mg T&E (WMD, 3.25 [95% CrI, 0.37–6.12]), IVA 2 mg monthly (WMD, 3.39 [95% CrI, 0.41–6.38]), IVBeva 1.25 mg monthly (WMD, 2.63 [95% CrI, 0.19–5.06]), and IVBeva 1.25 mg bimonthly (WMD, 3.79 [95% CrI, 0.84–6.79]) were associated with a statistically significant superiority in terms of mean change in BCVA. No statistically significant differences were seen among any other anti-VEGF monotherapies in the network. The SUCRA scores of the mean change in BCVA showed the rank of the result of relative effects (Figure 6), and the top five ranks of treatments were as follows: IVBeva 1.25 mg bimonthly (SUCRA = 80.2%), IVBeva 1.25 mg 3PRN (SUCRA = 80.1%), IVA 2 mg monthly (SUCRA = 79%), IVR 0.5 mg T&E (SUCRA = 76%), and IVR 0.5 mg monthly (SUCRA = 74%).

### 3.3 The Number of SAEs

Fifteen trials comparing fourteen interventions in terms of the number of SAEs were evaluated (Martin et al., 2011; El-Mollayess et al., 2012; Heier et al., 2012; Lushchyk et al., 2013; Wykoff et al., 2015; Schauwvlieghe et al., 2016; Feltgen et al., 2017; Silva et al., 2018; Amarakoon et al., 2019; Dugel et al., 2019; Gillies et al., 2019; Kertes et al., 2019; Wang et al., 2019). The VIEW 1 and VIEW 2 trials did not report the total number of SAEs; thus, only serious systemic adverse events in the two trials were analyzed. Figure 2 shows the results of the network meta-analysis. Compared with IVBeva 1.25 mg 1PRN, IVR 0.5 mg monthly (RR, 0.70 [95% CrI, 0.53–0.93]), IVA 2 mg monthly (RR, 0.62 [95% CrI, 0.42–0.94]), IVBeva 1.25 mg q6 weekly (RR, 0.28 [95% CrI, 0.09–0.72]), IVBeva 1.25 mg bimonthly (RR, 0.49 [95% CrI, 0.24–0.96]), and IVBro 3 mg q12w/q8w (RR, 0.56 [95% CrI, 0.34–0.92]) were associated with a statistically significant lower risk of SAEs. Compared with IVR 0.5 mg 1PRN, IVBeva 1.25 mg q6 weekly (RR, 0.37 [95% CrI, 0.12–0.96]) was associated with a significantly lower risk of SAEs. No statistically significant differences were seen among any other anti-VEGF monotherapies in the number of SAEs. The SUCRA scores of the number of SAEs showed the rank of the result of relative effects (Figure 6.), and the top five ranks of treatments were as follows: IVB 1.25 mg q6 weekly (SUCRA = 91%), IVBeva 1.25 mg bimonthly (SUCRA = 74%), IVBro 3 mg q12w/q8w (SUCRA = 72%), IVA 2 mg monthly (SUCRA = 62%), and IVBro 6 mg q12w/q8w (SUCRA = 58%). It is noted that the results of IVB 1.25 mg q6 weekly versus other competing interventions were wide-ranging because the relevant trials had small numbers of events and sample sizes.

### 3.4 The Proportion of Patients Who Gained ≥15 Letters in BCVA

**FIGURE 3:**
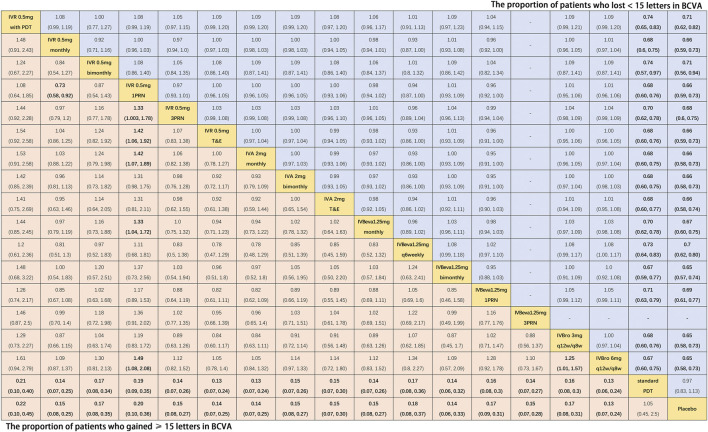
Pairwise comparisons of the network meta-analyses. The risk ratio (95% CrI) for comparisons is in cells in common between column-defining and row-defining treatments. Bold cells are significant. For the proportion of patients who gained ≥15 letters in BCVA, a risk ratio <1 favors column-defining treatment. For the proportion of patients who lost <15 letters in BCVA, a risk ratio <1 favors row-defining treatment.

Twenty-four trials (Heier et al., 2006; Rosenfeld et al., 2006; Brown et al., 2009; Martin et al., 2011; El-Mollayess et al., 2012; Heier et al., 2012; Larsen et al., 2012; Krebs et al., 2013a; Busbee et al., 2013; Kodjikian et al., 2013; Lushchyk et al., 2013; Wykoff et al., 2015; Schauwvlieghe et al., 2016; Feltgen et al., 2017; Haga et al., 2018; Silva et al., 2018; Amarakoon et al., 2019; Dugel et al., 2019; Gillies et al., 2019; Kertes et al., 2019; Wang et al., 2019; Lopez Galvez et al., 2020) comparing eighteen interventions were included in the analysis of the proportion of patients who gained ≥15 letters in BCVA from the baseline, namely, visual benefit. Figure 3 shows the results of the network meta-analysis. Compared with placebo and standard PDT therapy, all anti-VEGF monotherapies were associated with clearly superior visual benefit. Compared with IVR 0.5 mg 1PRN, IVR 0.5 mg monthly (RR, 1.37 [95% CrI, 1.08–1.74]), IVR 0.5 mg 3PRN (RR, 1.33 [95% CrI, 1.01–1.78]), IVR 0.5 mg T&E (RR, 1.42 [95% CrI, 1.06–1.92]), IVA 2 mg monthly (RR, 1.42 [95% CrI, 1.07–1.89]), IVBeva 1.25 mg monthly (RR, 1.33 [95% CrI, 1.04–1.72]), and IVBro 6 mg q12w/q8w (RR, 1.49 [95% CrI, 1.08–2.08]) were associated with a statistically significant superiority of visual benefit. In addition, IVBro 6 mg was slightly more effective than IVBro 3 mg (RR, 1.25 [95% CrI, 1.01–1.57]) for visual benefit. No statistically significant differences were seen among any other anti-VEGF monotherapies in terms of visual benefit beyond 15 letters in the network. The SUCRA scores showed the rank of the results of relative effects (Figure 6.), and the top five ranks of treatments were as follows: IVBro 6 mg q12w/q8w (SUCRA = 83%), IVR 0.5 mg T&E (SUCRA = 76%), IVA 2 mg monthly (SUCRA = 75%), IVR 0.5 mg monthly (SUCRA = 69%), and IVBeva 1.25 mg 3PRN (SUCRA = 66%).

### 3.5 The Proportion of Patients Who Lost <15 Letters in BCVA

Nineteen trials (Heier et al., 2006; Rosenfeld et al., 2006; Brown et al., 2009; Martin et al., 2011; El-Mollayess et al., 2012; Heier et al., 2012; Larsen et al., 2012; Busbee et al., 2013; Lushchyk et al., 2013; Schauwvlieghe et al., 2016; Haga et al., 2018; Silva et al., 2018; Amarakoon et al., 2019; Dugel et al., 2019; Gillies et al., 2019; Kertes et al., 2019; Wang et al., 2019) comparing seventeen treatments were included in the analysis of the proportion of patients who lost <15 letters in BCVA from the baseline, namely, visual stability. Figure 3 shows the results of the network meta-analysis. Compared with placebo and standard PDT therapy, all anti-VEGF monotherapies were associated with clearly superior visual stability. However, no statistically significant differences were seen among all anti-VEGF monotherapies in terms of visual stability in the network. The SUCRA scores showed the rank of the results of relative effects (Figure 6.), and the top five ranks of treatments were as follows: IVBro 6 mg q12w/q8w (SUCRA = 76%), IVBro 3 mg q12w/q8w (SUCRA = 74%), IVA 2 mg monthly (SUCRA = 73.2%), IVA 2 mg bimonthly 72.8%), and IVBeva 1.25 mg bimonthly (SUCRA = 72.5%).

### 3.6 Mean Change in CRT

**FIGURE 4:**
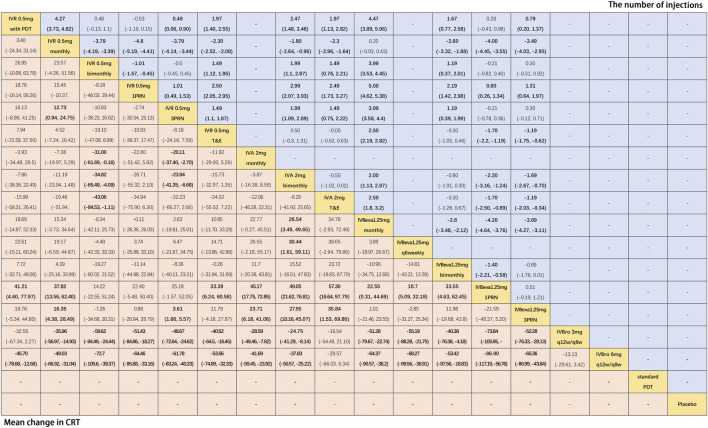
Pairwise comparisons of the network meta-analyses. Weighted mean differences (95% CrI) for comparisons are in cells in common between column-defining and row-defining treatments. Bold cells are significant. For the mean change in CRT from the baseline, weighted mean differences <0 favor row-defining treatment. For the mean number of injections, weighted mean differences <0 favor column-defining treatment.

Twenty-two trials (Martin et al., 2011; El-Mollayess et al., 2012; Larsen et al., 2012; Krebs et al., 2013a; Krebs et al., 2013b; Busbee et al., 2013; Kodjikian et al., 2013; Lushchyk et al., 2013; Wykoff et al., 2015; Schauwvlieghe et al., 2016; Weingessel et al., 2016; Feltgen et al., 2017; Haga et al., 2018; Silva et al., 2018; Amarakoon et al., 2019; Dugel et al., 2019; Gillies et al., 2019; Wang et al., 2019; Lopez Galvez et al., 2020) comparing sixteen interventions were included in the analysis of the mean change in CRT from the baseline. Figure 4 shows the results of the network meta-analysis. It is worth noting that IVBro q12w/q8w (both 3 and 6 mg) was associated with statistically significant superiority in decreasing CRT compared with the other anti-VEGF monotherapies, except IVA 2 mg T&E (WMD, −16.54 [95% CrI, −54.49, 21.10] and WMD, −29.57 [95% CrI, −66.03, 6.34]). Compared with IVB 1.25 mg 1PRN, except IVR 0.5 mg bimonthly, 1PRN, and 3PRN were associated with a statistically non-significant trend toward decreasing CRT, the other treatments were associated with clearly superiority in terms of mean change of CRT. Compared with IVR 0.5 mg bimonthly, IVA 2 mg with any therapeutic frequency (monthly (WMD, −31.00 [95% CrI, −61.69, −0.16]), bimonthly (WMD, −34.82 [95% CrI, −65.49, −4.09]), and T&E (WMD, −43.06 [95% CrI, −84.53, −1.11]), and IVBro with any therapeutic dosage (3 mg (WMD, −59.62 [95% CrI, −94.49, −24.44]) and 6 mg (WMD, −72.70 [95% CrI, −105.6, −39.37]) were associated with statistical superiority. Compared with IVBeva 1.25 mg 3PRN, IVA 2 mg with any therapeutic frequency (monthly (WMD, −23.71 [95% CrI, −41.08, −6.18]), bimonthly (WMD, −27.55 [95% CrI, −45.07, −10.16]), and T&E (WMD, −35.84 [95% CrI, −69.86, −1.53]), IVBro with any therapeutic dosage (3 mg (WMD, −52.28 [95% CrI, −76.33, −28.13]) and 6 mg (WMD, −65.36 [95% CrI, −86.99, −43.84]), IVR 0.5 mg monthly (WMD, -16.35 [95% CrI, −28.49, −4.38]), and 3PRN (WMD, −3.61 [95% CrI, −5.57, −1.66]) were associated with statistical superiority. Compared with IVR 0.5 mg 3PRN, IVA 2 mg monthly (WMD, −20.11 [95% CrI, −37.40, −2.70]) and bimonthly (WMD, −23.94 [95% CrI, −41.35, −6.68]) and IVR 0.5 mg monthly (WMD, −12.73 [95% CrI, −24.75, −0.94]) were associated with statistical superiority. In addition, IVA 2 mg bimonthly was associated with statistically significant superiority compared with IVBeva 1.25 mg monthly (WMD, 26.54 [95% CrI, 3.49–49.65]) and q6 weekly (WMD, 30.44 [95% CrI, 1.61–59.11]). No statistically significant differences were seen among any other anti-VEGF monotherapies in terms of the mean change in CRT in the network. However, the mean change in CRT versus other outcomes was generally wide-ranging. The SUCRA scores showed the rank of the results of relative effects (Figure 6.), and the top five ranks of treatments were as follows: IVBro 6 mg q12w/q8w (SUCRA = 99%), IVBro 3 mg q12w/q8w (SUCRA = 92%), IVA 2 mg T&E (SUCRA = 80%), IVA 2 mg bimonthly (SUCRA = 76%), and IVA 2 mg monthly (SUCRA = 70%).

### 3.7 The Mean Number of Injections

Fourteen trials (Martin et al., 2011; Larsen et al., 2012; Krebs et al., 2013a; Krebs et al., 2013b; Busbee et al., 2013; Kodjikian et al., 2013; Weingessel et al., 2016; Feltgen et al., 2017; Haga et al., 2018; Silva et al., 2018; Amarakoon et al., 2019; Gillies et al., 2019; Kertes et al., 2019; Lopez Galvez et al., 2020) comparing twelve treatments were included in the analysis of the mean number of injections. Figure 4 shows the results of the network meta-analysis. The monthly frequency (both IVR and IVBeva) was associated with more injections than other treatments. Compared with IVR 0.5 mg 1PRN, the other anti-VEGF monotherapies were associated with significantly more injections. PDT could partly reduce the mean number of anti-VEGF injections. The PRN frequency (both 1PRN and 3PRN) was generally associated with fewer injections than other treatments. The injection number of IVR 0.5 mg 3PRN was equal to IVR 0.5 mg bimonthly and associated with fewer injections than the other fixed injection frequency and T&E regimens. The SUCRA scores showed the rank of the results of relative effects (Figure 6.), and the top five ranks of treatments were as follows: IVR 0.5 mg 1PRN (SUCRA = 99%), IVR 0.5 mg with PDT (SUCRA = 89%), IVBeva 1.25 mg 1PRN (SUCRA = 79%), IVR 0.5 mg 3PRN (SUCRA = 69.7%), and IVR 0.5 mg bimonthly (SUCRA = 69.4%).

### 3.8 Additional Analysis

#### 3.8.1 Network Meta-Analysis of Anti-VEGF Drugs Regardless of Therapeutic Frequency

**FIGURE 5:**
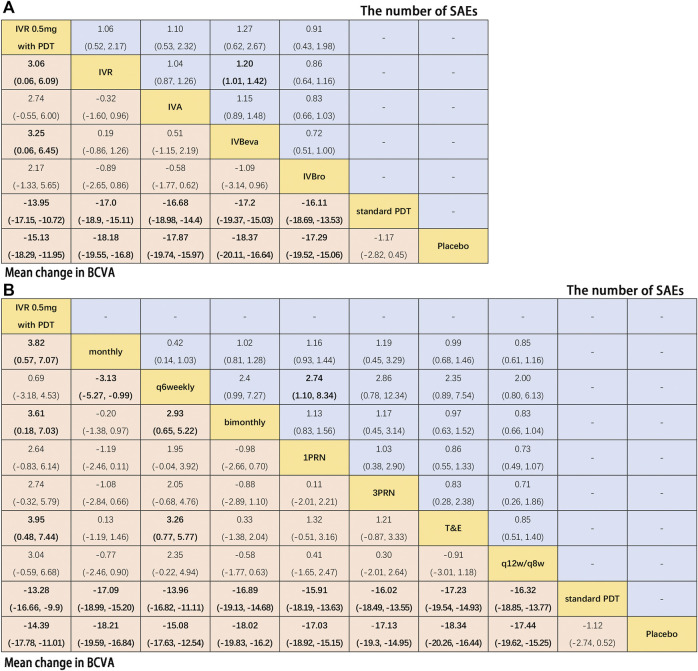
Pairwise comparisons of the network meta-analyses. **(A)** Anti-VEGF drugs regardless of therapeutic frequency. **(B)** Therapeutic frequency regardless of different anti-VEGF drugs. Comparison of the included interventions: Weighted mean differences (95% CrI) are for the mean change in BCVA, and risk ratios (95% CrI) are for the number of SAEs. Bold cells are significant. For the mean change in BCVA, weighted mean differences <0 favor column-defining treatment. For the number of SAEs, a risk ratio <1 favors column-defining treatment.

**FIGURE 6:**
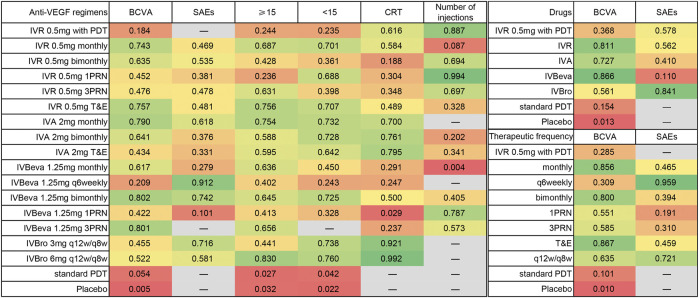
Surface under the cumulative ranking (SUCRA) based on each outcome. Higher SUCRA scores correspond to a higher probability of a treatment being in the top ranks.

Additional analysis was conducted to investigate the comparative efficacy and safety of individual anti-VEGF drugs (Figure 5). In terms of efficacy, sixteen trials were included in the analysis of the mean change in BCVA at 12 months from the baseline, comparing six interventions: IVR 0.5 mg with PDT, IVR, IVA, IVBeva, IVBro, standard PDT, and placebo. Compared with standard PDT and placebo, all four anti-VEGF interventions were associated with a statistically significant superiority in visual acuity. Comparing IVR 0.5 mg with PDT, IVR (WMD, 3.06 [95% CrI, 0.06–6.09]) and IVBeva (WMD, 3.25 [95% CrI, 0.06–6.45]) were associated with statistical superiority. It is worth noting that there were no statistically significant differences among the four anti-VEGF drugs (IVR, IVA, IVBeva, and IVBro) in the network. In terms of safety, ten trials were included in the analysis of the number of SAEs, comparing IVR 0.5 mg with PDT, IVR, IVA, IVBeva, and IVBro. IVR was associated with a significantly lower risk of SAEs than IVBeva (RR, 1.20 [95% CrI, 1.01–1.42]). There were no statistically significant differences among the other interventions. However, IVBro was associated with a statistically non-significant lower risk of SAEs than other anti-VEGF drugs. The SUCRA scores are shown in Figure 6.

#### 3.8.2 Network Meta-Analysis of Therapeutic Frequency Regardless of the Different Drugs

Further analysis was also conducted to investigate the comparative efficacy and safety of the therapeutic frequency of the anti-VEGF regimens (Figure 5). In terms of efficacy, twenty-two trials were included in the analysis of the mean change in BCVA at 12 months from the baseline, comparing ten interventions: IVR 0.5 mg with PDT, monthly, q6 weekly, bimonthly, 1PRN, 3PRN, T&E, q12w/q8w, standard PDT, and placebo. Compared with standard PDT and placebo, all treatment frequencies were associated with a statistically significant superiority of visual acuity. Compared with IVR 0.5 mg with PDT, monthly (WMD, 3.82 [95% CrI, 0.57–7.07]), bimonthly (WMD, 3.61 [95% CrI, 0.18–7.03]), and T&E (WMD, 3.95 [95% CrI, 0.48–7.44]) were associated with statistical superiority. Compared with q6 weekly, monthly (WMD, 3.13 [95% CrI, 0.99–5.27]), bimonthly (WMD, 2.93 [95% CrI, 0.65–5.22]), and T&E (WMD, 3.26 [95% CrI, 0.77–5.77]) were associated with a statistically significant superiority of visual acuity. No statistically significant differences were seen among any other therapeutic frequencies in the network. In terms of safety, thirteen trials were included in the analysis of the number of SAEs comparing monthly, q6 weekly, bimonthly, 1PRN, 3PRN, T&E, and q12w/q8w therapeutic frequencies. Q6 weekly was associated with a statistically non-significant lower risk of SAEs than other therapeutic frequencies, except 1RPN frequency (RR, 2.74, [95% CrI, 1.10–8.34]) and q12w/q8w showed relatively good safety. The SUCRA scores are shown in Figure 6.

### 3.9 Quality of Evidence

The bias assessment for eligible RCTs included in the network meta-analysis suggested no severe risk of bias (Supplementary Figures S2, S3 in Supplementary Material).

The results of the node-splitting analysis and *p* values are shown in the Supplementary Material. Most *p* values were larger than 0.05, which demonstrated no statistical inconsistencies between direct and indirect comparisons among any outcomes in any closed loop.

## 4 Discussion

The efficacy and safety of anti-VEGF agents have been shown in several RCTs and real-world studies. Since the first approval of ranibizumab in 2006, anti-VEGF agents have become the regular care for nAMD patients (Schmidt-Erfurth et al., 2014; Flaxel et al., 2020). Even though all anti-VEGF agents are based on VEGF inhibition, the different molecular architectures, sizes, and pharmacokinetic characteristics of anti-VEGF agents with different therapeutic dosages and frequencies may result in different efficacies and safety and should not be considered one entity. Given that a comprehensive efficacy and safety profile and the optimal therapeutic regimen for anti-VEGF monotherapies remain to be clearly defined, we included 27 head-to-head RCTs (10,484 patients) in the network meta-analysis of comparative efficacy and safety of anti-VEGF monotherapies for patients with nAMD.

This network meta-analysis showed that ranibizumab, bevacizumab, brolucizumab, and aflibercept with different therapeutic regimens were more effective in visual acuity than standard PDT and placebo, embodying the mean change in BCVA from the baseline, visual benefits, and stability maintenance. No significant differences were seen among the four anti-VEGF agents for visual efficacy, regardless of therapeutic regimens, suggesting that the four drugs have similar efficacy for visual acuity. However, concerning specific anti-VEGF regimens, statistically significant differences and statistically non-significant trends were observed. Comparatively, IVA 2 mg monthly and bimonthly, IVR 0.5 mg T&E and monthly, IVBeva 1.25 mg bimonthly, and IVBro 6 mg q12w/q8w were associated with a superior trend of visual efficacy. Notably, the sample size of IVBeva 1.25 mg bimonthly and q6 weekly was relatively smaller than that of other interventions, which might reduce the precision of the data. Regarding therapeutic frequency alone, the visual efficacy of T&E, monthly, and bimonthly regimens showed a statistically significant superiority versus q6 weekly and a better trend than PRN regimens, suggesting that proactive regimens might be superior to reactive regimens in visual efficacy. Comprehensively, IVA 2 mg bimonthly, IVR 0.5 mg T&E, and IVBro 6 mg q12w/q8w regimens were associated with better visual efficacy *via* relatively fewer therapeutic frequencies with a higher quality of evidence.

Furthermore, there were differences in the SAE numbers among the four anti-VEGF agents and specific therapeutic regimens. Brolucizumab (both 3 and 6 mg) generally showed a statistically non-significant trend of better safety than the other three anti-VEGF agents; moreover, the therapeutic frequency of brolucizumab, namely, the q12w/q8w and T&E regimens also presented a trend of lower risk of fewer SAEs. In addition, IVBeva 1.25 mg 1PRN showed a relatively higher risk of SAEs, which was derived from a CATT study that compared ranibizumab and bevacizumab (Martin et al., 2011). When merging different frequencies of anti-VEGF agents, ranibizumab showed a statistically significant trend of a lower risk of SAEs than bevacizumab. However, it was unaccountable that IVBeva 1.25 mg q6 weekly and bimonthly and the q6 weekly regimens showed a significantly lower risk of SAEs and even showed a significant difference compared with IVBeva 1.25 mg 1PRN, which needed large sample sizes to provide adequate power for a precise evaluation of the safety outcomes (El-Mollayess et al., 2012; Lushchyk et al., 2013). Plyukhova et al. compared the safety of bevacizumab, ranibizumab, and aflibercept and reported that systemic adverse events were significantly higher in bevacizumab than in ranibizumab. The other adverse events, both systemic and ocular, did not differ significantly (Plyukhova et al., 2020). In addition, ocular serious adverse events were not included in our analysis. The rarity of ocular SAE made it impossible to identify even existing differences in the case that it does take place, and it impacted potential publication bias. It is worth noting that the systemic safety profile of brolucizumab is favorable and that the rates of cardiovascular or cerebrovascular events are typically low, but one of the main ocular adverse events is intraocular inflammation (IOI), which is related to severe visual acuity loss associated with retinal vasculitis and retinal occlusive vasculitis (Baumal et al., 2020). In the post hoc review of HAWK and HARRIER, the incidence of IOI in brolucizumab-treated eyes can be interpreted as at least 4.6% (vs. 1.1% for aflibercept-treated eyes) (Monés et al., 2021). Thus, vigilance in practice with active surveillance of IOL cases is encouraged, and physicians need to balance these risks against the efficacy and durability of brolucizumab in nAMD patients.

Notably, there were significant differences in the mean change in CRT among specific anti-VEGF regimens. Brolucizumab (both 3 and 6 mg) generally showed clearly superior efficacy of anatomical structures compared with other anti-VEGF regimens, supporting the hypothesis that a lower molecular weight with a high concentration gradient might increase the drug distribution to the target site, further controlling the anatomic lesion activity more effectively (Dugel et al., 2019), except IVA 2 mg T&E, which also had a relatively superior representation. The PRN regimens of ranibizumab were associated with a relatively inferior trend of reducing CRT versus brolucizumab and aflibercept. Bevacizumab was generally associated with an inferior trend toward reducing CRT, including monthly, weekly q6, and especially 3PRN and 1PRN, which showed statistically significant differences compared with the two regimens of brolucizumab, three regimens of aflibercept, and IVR monthly.

Focusing on the mean number of injections over 12 months, significant differences were reached between the monthly regimens versus the other regimens. Flexible regimens, such as those with dosing as needed PRN or T&E, were evaluated to reduce the retreatment frequency and monitoring burden, which might lessen the increased rate of geographic atrophy caused by monthly injections (Martin et al., 2011; Silva et al., 2018). The PRN regimens (both 1PRN and 3PRN) were associated with fewer injections than other regimens, especially ranibizumab 1PRN, which had the fewest number of injections. The reactive regimens were associated with relatively fewer injections versus proactive regimens (T&E and bimonthly), and statistically significant differences and statistically non-significant trends were observed. The q12w/q8w regimen identified a suitable maintenance dose interval based on individual treatment needs, driven by disease activity. The aim of regimen choice was to achieve favorable efficacy, effective treatment scheduling, and minimal monitoring burden.

Even though the main pharmacological targets of the three anti-VEGF agents are the same, namely, all isoforms of VEGF-A, the drug structure and the type and weight of molecules are different. Therefore, the pharmacological mechanism and pharmacokinetic profile of the three anti-VEGF agents may be different, in turn impacting the risk or benefit ratio. VEGF plays a crucial role in angiogenesis by activating VEGF receptors. Analysis of the interaction of VEGFA with the binding domains of antiangiogenic agents revealed that the aflibercept/VEGFA complex was stabilized by electrostatic force, whereas ranibizumab and the bevacizumab/VEGFA complex were characterized by van der Waals energy stabilization. The high stabilizing electrostatic energy of aflibercept may be attributed to its high association rate with VEGFA, whereas the relatively low experimental dissociation rate of ranibizumab may be due to lower conformational fluctuations, a higher number of contacts, and hydrogen bonds of the ranibizumab/VEGFA complex (Platania et al., 2015). Thus, the molecular interactions and stabilizing energy of these anti-VEGF agents are significantly different. In terms of binding manner, the different VEGF binding manners may contribute to different binding stoichiometry, Fcγ receptor affinity, propensity of platelet activation, and ability to bind epithelial and endothelial cells *in vitro*. Aflibercept forms a homogenous 1:1 complex with each VEGF dimer, which does not increase affinity for the low-affinity Fcγ receptor and does not activate platelets (Macdonald et al., 2016). These factors may be associated with the safety and adverse events of anti-VEGF agents. To compensate for these limitations of existing anti-VEGF agents, novel anti-VEGF molecules as potential candidate drugs should be developed to explore a stronger and more durable antiangiogenic efficacy. The preclinical animal models for testing efficacy include laser-induced choroidal neovascularization and oxygen-induced retinopathy modalities that can be applied for preclinical tests followed by further clinical trials (Hong et al., 2020).

Most notably, our study overcomes the shortcomings of the current network meta-analyses. First, we restricted the drugs and dosage strictly and only chose the recommended dosages of ranibizumab and aflibercept by the newest guidelines. Pegaptanib, ranibizumab 0.3 mg, and aflibercept 0.5 mg have been proven to have poor efficacy and are not recommended. Therefore, it was unnecessary to include drugs and dosagesthat are not recommended, and it was erroneous to merge different dosages into one group, such as merging 0.3 and 0.5 mg ranibizumab and merging 0.5 and 2 mg aflibercept, which might lead to inaccurate data and high heterogeneity (Ye et al., 2020). Similarly, the indiscriminate and non-standard merger of different therapeutic frequencies caused inaccurate results, such as combining different PRN regimens (Ye et al., 2020). Second, we selected the study strictly and collected data standardly. The errors of study selection and data collection would cause a serious mistake. Conbercept was included in the previous study (Ye et al., 2020), which actually could not be included due to the lack of RCTs available to connect to the node graph of the network due to the lack of true placebo groups in the PHOENIX and AURORA studies to form closed loops for the network and the reassigned regimen (Li et al., 2014; Liu et al., 2019). Similarly, Dugel’s study (2017) of brolucizumab was included in the previous study non-standardly due to different therapeutic frequencies and week 40 available data only (Dugel et al., 2017; Ye et al., 2020). Third, to maximize the clinical significance and acquire evidence with as high quality as possible for our study, we included anti-VEGF monotherapies that were recommended by current guidelines only, without various combination treatments, such as NSAIDs, triamcinolone, and radiation therapy, which might have no value for the current clinical applications (Zhao et al., 2021). We only included IVR 0.5 mg with PDT and standard PDT therapy to provide a more complete network closed loop to obtain more indirect evidence.

Our study held its own strengths and limitations. The strength of our study was the systematic methods of studies and data retrieval *via* PRISMA-NMA guidelines, comprehensive inclusion of outcomes, and Cochrane risk of bias tool usage. However, several limitations in our present study merit further discussion. Regarding the limitation of the meta-analysis of aggregate data, rather than individual patient data, investigations of potential heterogenicity, such as ethnic, regional, economic, and medical differences, were difficult. In the different RCTs, the baseline vision criteria for patient inclusion may be different, the retreatment criteria may be different, and the included lesion types may not be the same. Furthermore, we excluded a few RCTs that presented visual outcomes in non-transferable forms or studies with combined results, such as the IVAN trial (Chakravarthy et al., 2012). On the other hand, safety issues considering ocular serious adverse events were not included in our analysis. It was limited by several RCTs not specifically reporting ocular SAE and no sufficient data available to connect to the node graph of our network. Finally, our analysis only included the treatment regimens and results from RCTs conducted under experimental conditions with tightly defined treatments and did not include real-life studies that usually better reflect the routine clinical practice. This choice elevated the quality of the evidence relatively and can be applied to settings in which intensive treatment regimens were implemented, whereas it did not fully reflect the posology used in real clinical practice and limited the practice application in real-life surroundings in which undertreatment was common. Therefore, potential differences in relative efficacy and safety might exist among these anti-VEGF regimens in real-world settings.

Above all, balancing benefits and relative risks for each regimen is a crucial issue in choosing nAMD regimen options in clinical practice. Comprehensive evidence suggested that aflibercept bimonthly, ranibizumab T&E, and brolucizumab 6 mg q12w/q8w regimens had better visual efficacy than the other regimens. Brolucizumab had absolute superiority in anatomical recovery of the retina and a relative advantage of safety, as well as good performance of aflibercept T&E. The proactive regimens (T&E and bimonthly) had slightly better efficacy but a slightly increased number of injections versus the reactive regimen (PRN), which could be considered for real-world burdens. Bevacizumab had a statistically non-significant trend toward a lower degree of efficacy and safety than the other three anti-VEGF agents. Balancing benefits and relative risks and relieving various burdens seem mandatory to obtain maximal benefit from anti-VEGF monotherapies. In the current landscape, based on the premise of equivalent efficacy and safety, the optimal choice of anti-VEGF monotherapies may have the potential to improve the quality of vision, minimize SAEs, and maximize patient outcomes.

## 5 Conclusion

This network meta-analysis compared the efficacy and safety among the four anti-VEGF agents with various therapeutic regimens in terms of visual and anatomical efficacy, SAEs, and the number of injections, suggesting that brolucizumab 6 mg q12w/q8w, aflibercept 2 mg bimonthly or T&E, and ranibizumab 0.5 mg T&E are the ideal anti-VEGF regimens for nAMD patients. In the current landscape, based on the premise of equivalent efficacy and safety, the optimal choice of anti-VEGF monotherapies seems mandatory to obtain maximal benefit.

## Data Availability

The original contributions presented in the study are included in the article/Supplementary Material; further inquiries can be directed to the corresponding authors.
