# Supplementary material for: Efficacy and Safety of Anti–Vascular Endothelial Growth Factor Monotherapies for Neovascular Age-Related Macular Degeneration: A Mixed Treatment Comparison
Source: Front Pharmacol. 2021 Dec 21;12:797108. doi: 10.3389/fphar.2021.797108 (PMC8724554; doi:10.3389/fphar.2021.797108)

Supplementary Material

# Table S1. Literature Search Strategy (April 16th，2021)

| PubMed | Embase | Cochrane library |
| --- | --- | --- |
| #1 ((((((((Macular Degenerations) OR age-related macular degeneration) OR "Age Related Maculopathy") OR "Age Related Maculopathies") OR "age related macular degenerations") OR "Macular Dystrophy") OR Macular Dystrophies)) OR ((AMD) OR ARMD)  #2 "ranibizumab"[MeSH Terms] OR "ranibizumab"[All Fields] OR "lucentis"[All Fields]  #3 (BROLUCIZUMAB) OR RTH258  #4 "bevacizumab"[MeSH Terms] OR "bevacizumab"[All Fields] OR "avastin"[All Fields]  #5 "aflibercept"[Supplementary Concept] OR "aflibercept"[All Fields]  #6 "KH902 fusion protein"[Supplementary Concept] OR "KH902 fusion protein"[All Fields] OR "conbercept"[All Fields]  #7 angiogenesis inhibitors  #8 (verteporfin) OR Photodynamic therapy  #9 #2 OR #3 OR#4 OR #5 OR #6 OR #7 OR #8 OR #9  #10 (((((((("Randomized Controlled Trial" [Publication Type]) OR "Controlled Clinical Trial" [Publication Type]) OR "randomized" [tiab]) OR "placebo" [tiab]) OR "Clinical Trials as Topic"[Mesh:NoExp]) OR "randomly" [tiab]) OR "trial" [ti])) NOT (("Animals" [mh]) NOT " humans" [mh])  #11 #1 AND #9 AND #10 | 1. macular degeneration.mp. or exp retina macula age related degeneration/ or exp macular degeneration/ or exp retina macula degeneration/  2. ranibizumab.mp. or exp ranibizumab/  3. bevacizumab.mp. or exp bevacizumab/  4. aflibercept.mp. or exp aflibercept/  5. conbercept.mp. or exp conbercept/  6. Photodynamic therapy.mp. or exp photodynamic therapy/  7. verteporfin.mp. or exp verteporfin/  8. BROLUCIZUMAB.mp. or brolucizumab/  9. 2 or 3 or 4 or 5 or 6 or 7 or 8  10. random:.tw. or placebo:.mp. or double-blind:.tw.  11. 1 and 9 and 10 | #1 MeSH descriptor: [Macular Degeneration] explode all trees  #2 (Macular Dystrophies):ti,ab,kw (Word variations have been searched)  #3 (age related macular degeneration):ti,ab,kw (Word variations have been searched)  #4 (Age Related Maculopathy):ti,ab,kw (Word variations have been searched)  #5 (ARMD):ti,ab,kw (Word variations have been searched)  #6 (AMD):ti,ab,kw (Word variations have been searched)  #7 #1 or #2 or #3 or #4 or #5 or #6  #8 (ranibizumab):ti,ab,kw (Word variations have been searched)  #9 (bevacizumab):ti,ab,kw (Word variations have been searched)  #10 (aflibercept):ti,ab,kw (Word variations have been searched)  #11 (conbercept):ti,ab,kw (Word variations have been searched)  #12 (verteporfin):ti,ab,kw (Word variations have been searched)  #13 (BROLUCIZUMAB):ti,ab,kw (Word variations have been searched)  #14 (RTH258):ti,ab,kw (Word variations have been searched)  #15 MeSH descriptor: [Photochemotherapy] explode all trees  #16 (Photodynamic therapy):ti,ab,kw (Word variations have been searched)  #17 #8 or #9 or #10 or #11 or #12 or #13 or #14 or #15 or #16  #18 #7 and #17 |

# Figure S1. PRISMA flow diagram of eligible studies.


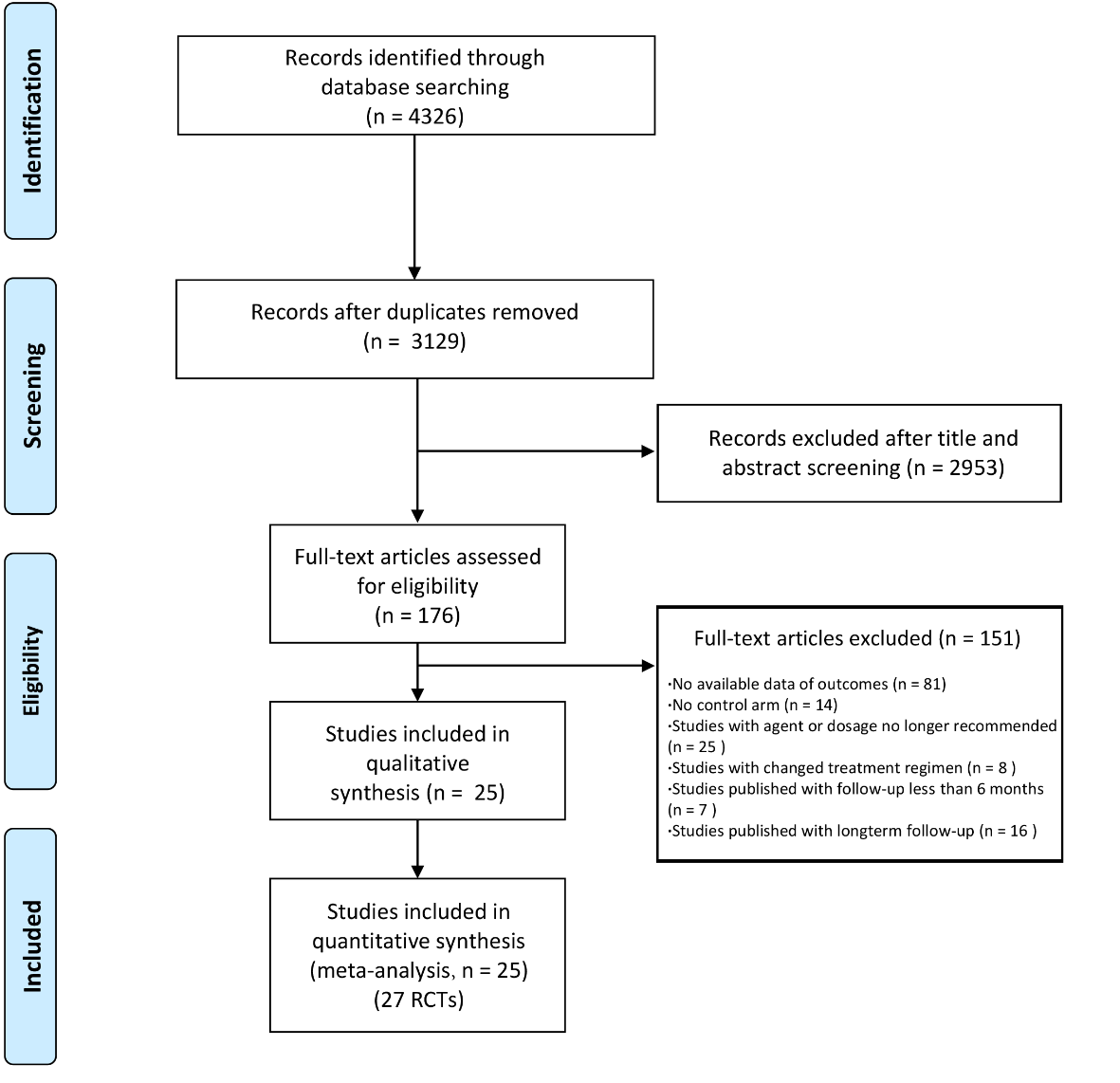


# Figure S2. Risk of bias graph


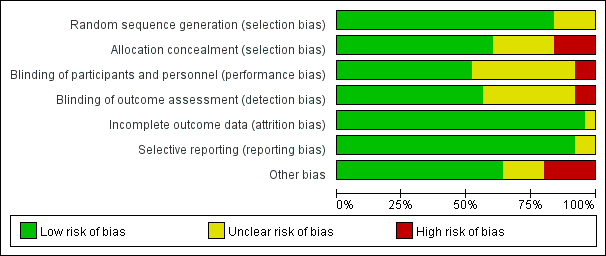


# Figure S3. Risk of Bias summary


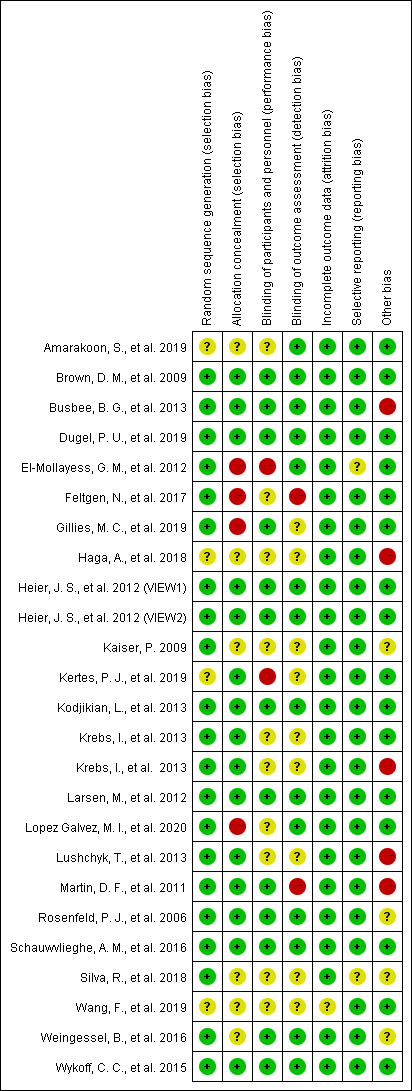

Supplement: Supplementary file 1 [file Table1.DOCX]
